# Teaming up to prevent foodborne disease.

**DOI:** 10.3201/eid0707.017712

**Published:** 2001

**Authors:** A P Liang, M Koopmans, M P Doyle, D T Bernard, C E Brewer

**Affiliations:** Centers for Disease Control and Prevention, Atlanta, Georgia 30333, USA. aliang@cdc.gov


### Teaming Up To Prevent Foodborne Disease

**Arthur P. Liang,* Marion Koopmans,† Michael P. Doyle,‡ Dane T. Bernard,§ and Camille E. Brewer¶**

*Centers for Disease Control and Prevention, Atlanta, Georgia, USA; †National Institute of Public Health and the Environment, Bilthoven, The Netherlands; ‡University of Georgia, Athens, Georgia, USA; §National Food Processors Association, Washington, DC, USA; and ¶Food and Drug Administration, Washington, DC, USA

Preventing foodborne disease requires the efforts of different segments of our "global" society. This session featured presentations on emerging issues across the spectrum of food safety activities, from science and technology to policy development and regulation.

### Comprehensive Surveillance of Human Caliciviruses in The Netherlands

Norwalk-like caliciviruses (NLV) are increasingly recognized as a significant cause of food-related illness. In recent years, The Netherlands has conducted four NLV studies: i) a physician-based case-control study, 1996-99; ii) a population-based cohort study with a nested case-control design, 1999; iii) NLV testing of stools obtained from patients who became ill during gastroenteritis outbreaks reported through municipal health services; and iv) NLV testing in samples from animal surveillance systems. NLVs were the causative agent in 5% of patients with foodborne illness seen by a general practitioner and 16% of patients in the community cohort. In the past 6 years, NLVs have caused more than 80% of all gastroenteritis outbreaks. Multiple NLV variants cocirculate in the population, and their diversity allows researchers to trace outbreaks to a common source. Highly related viruses have been found in herds of calves and swine, suggesting that animals may be a reservoir. A project has been initiated to study transmission patterns of these viruses across Europe.

### Reducing the Risk of Pathogens in Foods

Under Hazard Analysis and Critical Control Point (HACCP) systems, food manufacturers identify points where contamination is likely to occur and implement process controls to prevent it. A current limitation of the HACCP is that very few CCP practices are available for on-farm use, although several interventions appear to have promise. These practices include probiotic bacteria (benign bacteria that can be used to out-compete pathogenic bacteria) that prevent colonization by pathogens, edible vaccines that stimulate IgA production in an animal's gut, and improved farm management practices, such as reducing pathogen contamination in watering systems. Food processors have a larger array of current and promising control measures available, but most measures have limitations. For example, irradiation may cause undesirable "off" flavors in meat and poultry or undesirable texture characteristics in vegetables such as lettuce.

Address for Correspondence: Arthur P. Liang, Centers for Disease Control and Prevention, MS G24, Atlanta, GA 30333 USA; fax: 404-639-2212; e-mail: aliang@cdc.gov

Some treatments such as probiotic bacteria may be especially useful for protecting high-risk populations, such as the immunocompromised. Inevitably, consumers must also play a role in preventing foodborne disease. Consumers must be more active in such practices as avoiding undercooked and uncooked high-risk foods, refrigerating perishable foods, and disposing of hazardous foods that have been recalled.

### Science as Basis for Regulations

The food industry bears responsibility for providing safe foods for consumption. A framework of laws, regulations, and an inspection system facilitates production of safe foods. Food safety regulations fall into two basic classes: process-based and performance-based. Future legislative and regulatory requirements will focus more on performance-based standards, leaving the specifics of how the standard is achieved to individual processors, although most will likely develop HACCP programs. Future regulations, however, will also consider international agreements, such as those that enable the World Trade Organization to impose obligations that nations must fulfill to enter into free trade.

Both international and domestic policy will increasingly rely on the discipline of risk analysis for decision-making. The need for better data to conduct risk assessment will spur increased emphasis on foodborne illness surveillance systems like FoodNet (1) and PulseNet (2). Results of risk assessments must be analyzed through the risk management process to yield food safety policies that lead to development of performance criteria that the food industry can use to develop safer food processes.

### Borders? What Borders?

There has been a globalization of the food supply. Although no evidence supports the idea that imported food is less safe than domestic food, the Food and Drug Administration's (FDA) imported foods plan recognizes that some food safety issues, such as lack of regulatory authority and basic infrastructure, are specific to developing nations. Along with important surveillance and sampling activities, FDA provides international training and fosters technical cooperation aimed at prevention at the source of production. FDA's international partners are often from industry, nongovernment institutions, and universities. Other key partners include Food and Agricultural Organization, Pan American Health Organization, Instituto Interamericano de Cooperacion para la Agricultura, as well as the Centers for Disease Control and Prevention and the Foreign Agricultural Service. In 1999, FDA tested 1,000 samples of imported celery, cantaloupe, cilantro, green onions, parsley, loose-leaf lettuce, strawberries, and broccoli for *Salmonella*, *Shigella*, and *Escherichia coli* O157:H7. To date, 95.4% of these samples have been free of contamination. FDA has worked closely with several countries (e.g., Costa Rica, Trinidad and Tobago, Honduras, and Jamaica) to determine their needs and capacities. Examples of training and outreach include a Regional Outreach Meeting on Food Safety in Mexico City, Mexico, and one in Santiago, Chile.

To paraphrase The Future of Public Health, the 1988 Institute on Medicine report, food safety is not just what government regulators or industry quality assurance managers do. Food safety is what society does to ensure the conditions under which people can consume food that is safe, as well as wholesome and nutritious. Safe food requires the work of producers and consumers; industry and government; local, state, federal, and, increasingly, international partners.

### References

1. Mead PS, Slutsker L, Dietz V, McCaig LF, Bresee JS, Shapiro C, et al. Food-related illness and death in the United States. Emerg Infect Dis 1999;5:607-25.
2. Swaminathan B, Barrett TJ, Hunter SB, Tauxe RB, and the CDC PulseNet Task Force. PulseNet: the molecular subtyping network for foodborne bacterial disease surveillance, United States. Emerg Infect Dis 2001;7:382-9.

---

### Ethical and Legal Issues in Infectious Disease Research and Control

**Christine Grady,* Gita Ramjee,† Jean Pape‡**
**Karen Hofman,* and Majorie Speers§**

*National Institutes of Health, Bethesda, Maryland, USA; †Medical Research Council, Durban, South Africa; ‡Group Haitien d'Etude du Sarcome de Kaposi et des Infections Opportunistes, Port-au-Prince, Haiti; §Centers for Disease Control and Prevention, Atlanta, Georgia, USA

Ethical issues in international research have received an increasing amount of publicity over the past decade, as more attention is focused on global health and expanded funding is provided for research in the developing world. Much of the discussion has focused on issues that arise when researchers from countries rich in resources collaborate with researchers from poorer countries. Some of the controversies result from research on vaccines and drugs for emerging infectious diseases. This panel addressed ongoing challenges to conducting ethical research.

Christine Grady from the National Institutes of Health reviewed existing international codes and guidelines for conducting research. Ethical concerns in international research often stem from the potential for study communities in the developing world to be vulnerable to exploitation because of their social and economic circumstances. Several international codes provide guidance on the ethical conduct of clinical research including the Declaration of Helsinki, Council for International Organizations of Medical Sciences (CIOMS), International Guidelines for Biomedical Research, and the UNAIDS Guidance Document on Ethical Considerations in HIV Vaccine Research. However, these codes are recommendations, not legal imperatives. In addition, they do not address how disagreements might be resolved. Dr. Grady mentioned some current ethical dilemmas, including determination of treatment provided to participants during the course of a clinical trial, the obtaining of informed consent, obligations of researchers to the study community, and investigators' responsiveness to local health needs. Although there are no easy answers, she suggested that basic ethical principles should be applied globally, with local interpretation and implementation.

Gita Ramjee from the South African Medical Research Council discussed ethical problems and solutions used by South African researchers conducting a phase-III trial of a vaginal microbicide to prevent HIV infection. Researchers realized that participants did not understand the details of the study. Investigators then modified the process to include role playing and to allow time for trial participants to consult with peers. Researchers also added a knowledge testing component to the trial. These changes suggest that informed consent is not a one-time event but is instead an ongoing process.

The need to minimize the risk of participants' contracting HIV during the study necessitated an intensive counseling component. Participants were informed about local HIV counseling services, and sex workers were encouraged to set standard prices and to refuse clients who would not use condoms. This study shows how practical solutions can be used to reduce risks associated with research.

Finally, Jean Pape from Les Centres GHESKIO in Port-au-Prince, Haiti, highlighted the major challenges to conducting international research, which center on obtaining ethical review or institutional review board clearance. See page 547 for a more detailed article concerning this discussion.

Address for correspondence: Karen Hofman, Fogarty International Health Center, National Institutes of Health, 16 Center Drive, Building 16, Bethesda, MD 20892-6705, USA; fax: 301-594-1211; e-mail: hofmank@mail.nih.gov
